# Complete spontaneous regression of a total pneumothorax in a patient with chronic obstructive lung disease

**Published:** 2011-11-24

**Authors:** G Ugur, M Arzu, Y Muammer

**Affiliations:** Canakkale Onsekiz Mart University, School of Medicine, Department of Chest Diseases, 17100, CanakkaleTurkey

**Keywords:** Pneumothorax, secondary, chronic obstructive pulmonary disease, treatment

## Abstract

A 61-year old man presented with a sudden onset of breathlessness. The total left pneumothorax was overlooked on the initial chest radiograph. One month later, the patient had a partial pneumothorax less than 20% on the radiograph, although he did not receive any therapy against pneumothorax, such as oxygen inhalation or needle aspiration. After the observation for one month, the lungs totally expanded. Pulmonary function tests demonstrated severe chronic obstructive pulmonary disease. To our knowledge, this is the first case in which the total secondary pneumothorax showed a spontaneous remission.

## Introduction

The aims of the therapy for pneumothorax are to evacuate the space, achieve closure of the leak, and prevent the recurrence. The categories of treatment methods are the simple observation with oxygen inhalation, aspiration, chest tube drainage, video-assisted thoracoscopic surgery, and thoracotomy. [**1**] Simple observation is generally reserved for asymptomatic patients with a small (less than 20%) unilateral pneumothorax. We present a case with total spontaneous pneumothorax showing complete resolution without any treatment.

## Case Presentation

**Fig 1:**
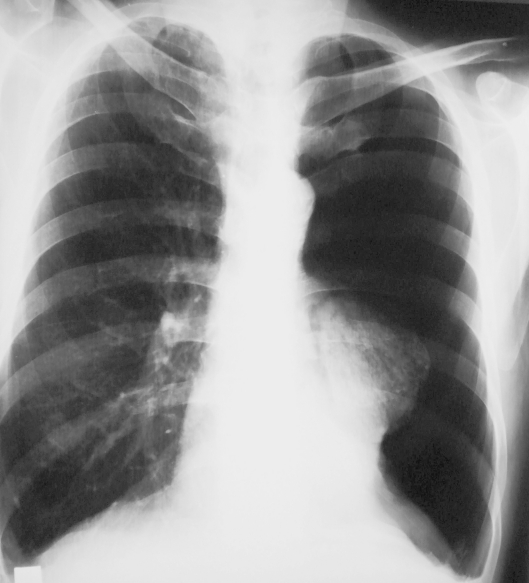
Chest x-ray showing an air density in left lung fields

A 61-year old man was admitted to the emergency department with complaint of acute breathlessness. After chest x-ray (Fig. 1), the patient was diagnosed with pneumonia in his left lung fields, and treated with antibiotics. One month later, the patient presented to our clinic with complaints of cough and sputum. 

**Fig. 2:**
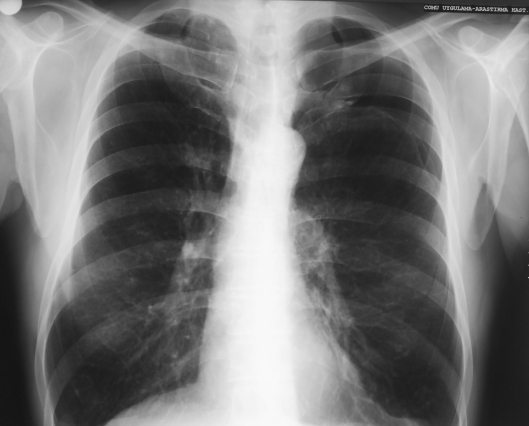
A left partial (20%) pneumothorax

Forced vital capacity (FVC) 2720 ml (62%), forced expiratory volume in one second (FEV1) 1370 ml (40%), and FEV1/FVC 50% in pulmonary function tests. The patient was diagnosed with severe chronic obstructive pulmonary disease and was treated with bronchodilator agents. One month later, both lungs were totally expanded and the recurrence was not observed for one year.

## Discussion

It has been noted that therapy for secondary pneumothorax should be more aggressive because of the higher rate of recurrence due to the underlying lung disease. [**1**] Observation therapy can be appropriate only for the patients who had minimal (less than 20%) unilateral pneumothorax but mortality rate can reach 5% for such cases due to tension pneumothorax during observation therapy. [**2**]

According to GOLD classification [**3**] the patient was categorized with severe chronic obstructive pulmonary disease (COPD). COPD is the most common cause of secondary pneumothorax. Cough is a common symptom in the course of COPD. The increase in intrathoracic pressure during cough can cause or augment pneumothorax. [**1**]

On the other hand, these patients have generally bleb or bulla due to airway obstruction. In contrast to these negative effects, our patient showed a well improvement without a therapy. This finding may be due to “higher” permeability of the pleural surfaces. In our knowledge, this is the first case in which secondary total pneumothorax showed a spontaneous remission.
